# Candida non albicans isolates with a high antibiotic resistance: a real threat for cancer patients in Karaj City

**DOI:** 10.1186/2047-2994-4-S1-P170

**Published:** 2015-06-16

**Authors:** E Kalantar, M Asadi, S Hatami, M Agha Barari, M Hadi Naseh, E Mahmoudi, K Kabir

**Affiliations:** 1 Department of Microbiology and Immunology, School of Medicine, Karaj, Iran, Islamic Republic Of; 2 Department of Oncology, Shahid Madani Hospital, Karaj, Iran, Islamic Republic Of; 3 School of Nursery and Midwifery, Alborz University of Medical Sciences, Karaj, Iran, Islamic Republic Of; 4 Deputy of Treatment, Alborz University of Medical Sciences, Karaj, Iran, Islamic Republic Of; 5 Department of Pathobiology, Alborz University of Medical Sciences, Karaj, Iran, Islamic Republic Of; 6 Department of Social Medicine, School of Medicine, Alborz University of Medical Sciences, Karaj, Iran, Islamic Republic Of

## Introduction

Cancer patients remain at substantial risk for developing serious infections particularly oropharyngeal Candidiasis. Because of a weakened line of defense in oral cancer patients, the present prospective study was carried out, with the aim of isolation, and identification of Candida spp from oral cavity of cancer patients.

## Methods

From 50 cancer patients with oropharyngeal candidiasis sample was taken by the physician. All samples were sent to Research Laboratory, School of Medicine, Alborz University of Medical Sciences and processed by standard methods for Isolation and identification. Antifungal resistance pattern was carried out according to CLSI guideline.

By Multiplex Polymerase Chain Reaction, identification of the 18s Ribosomal RNA among *Candida spp was* performed using specific primers for the molecular identification of *Candida spp*.

## Results

Of the 50 patients which, 18 (36%) were female and 32 (64%) were male; mean ages was 38.4 years. Leukemia and lymphoma were the most frequent cancer in the studied group, accounting for 17 (34%) and 12 (24%) respectively. The mean weight of the patients was 73.2 Kg. A total of 29 *Candida spp* were isolated from 29 of cancer patients with oropharyngeal candidiasis; of which 17 were *C. albicans* and 12 were *C. non – albicans*.

All the *Candida spp* were confirmed using the 18s Ribosomal RNA specific primers for the molecular identification of *Candida spp.* Among all the *Candida spp, Candida non – albicans* showed a high resistance pattern to amphotricin B (MIC 07 µg / ml) and ketokenazole (MIC=05 µg / ml).

## Conclusion

In conclusion, oropharyngeal Candidiasis is a serious infection among cancer patients. The isolated *candida spp* were resistant to common antifungal agents which may leads to longer hospital stay, more expensive/ toxic drugs and higher mortality. Therefore, interval surveillance is necessary in developing institutional guidelines.

## Disclosure of interest

None declared.

